# Estimation of the soil hydraulic properties from field data by solving an inverse problem

**DOI:** 10.1038/s41598-020-66282-5

**Published:** 2020-06-09

**Authors:** Lamia Guellouz, Brahim Askri, Jérome Jaffré, Rachida Bouhlila

**Affiliations:** 1 LMHE, ENIT, Université de Tunis El Manar, Tunis, Tunisia; 2 INRIA Paris, 2 rue Simone IFF, 75589 Paris, France

**Keywords:** Environmental sciences, Hydrology

## Abstract

Estimating unsaturated soil hydraulic properties to predict water dynamics through a vertical soil profile under the effects of irrigation, drainage and evapotranspiration is imperative for managing soils in the arid regions. The aim of this work was to determine the soil water retention curve and the hydraulic permeability function of a bare soil profile in a Tunisian oasis threatened by salinization. The developed model combines a numerical inversion of the unsaturated flow equation with the BOBYQA optimisation algorithm. The direct model solved the Richards equation using a cell-centred finite difference model. Hydraulic properties were described by van Genuchten-Mualem models. Input data for the inverse problem are the infiltration flow, soil water contents and pressure heads measured during ponded infiltration and internal drainage tests. Numerical simulations of these two tests were performed considering a homogeneous single-layer soil profile but a better fitting between measured and simulated water contents was obtained when the soil profile was divided into five sub-layers. The hysteresis phenomenon was highlighted from the soil water retention and the relative permeability curves corresponding to the ponded infiltration and internal drainage tests.

## Introduction

Soil salinization is a major problem affecting the crop productivity in several Tunisian oases^1^. The mismanagement of irrigation water and the lack of external drainage, combined with the capillary rise and evaporation processes, lead to the rise of the saline groundwater and therefore to the salt accumulation in the top soil layer^2^. The control of this problem requires a good understanding of the water dynamics and solute transport in the vertical soil profile under the effects of irrigation, drainage, evaporation and transpiration processes. Simulations of these processes can be achieved using unsaturated flow models such as HYDRUS-1D^3^ and SWAP^4^. These tools are able to predict the impact of irrigation with saline water on soil salinity and crop yield^5,6^. Their application requires the determination of the relationships between the soil water content and pressure head, i.e. the soil water retention curve, and between the soil hydraulic conductivity and pressure head. These two relationships are generally described with parametric models involving empirical and physicals parameters. The water retention function and hydraulic conductivity can be determined by laboratory or field methods. These methods are either expensive or difficult to carry out or not reliable. Indeed the values of theses coefficients, when determined in laboratory^7^, are not necessarily representative of the field conditions^8^. Actually, methods such as infiltration method^9^, internal drainage method^10^, and evaporation method^11–13^ can be used in the field to determine the soil hydraulic properties from easily measurable data of infiltration rate, soil moisture content and pressure head. However, the most of these methods is costly, especially when large areas have to be characterised^14^.

During the last two decades, several inverse problem methods were developed to estimate the soil hydraulic properties from transient flow events^15–19^. These methods were based on the numerical inversion of the unsaturated flow equation and the use of optimisation algorithms. In such methods, the unknown parameters are determined indirectly by minimising deviations between measured and simulated flow attributes; e.g. infiltration flow, soil volumetric water content or pressure head^20^. Despite the large number of calculation required, this approach has great potential to accommodate any combination of boundary and initial condition. However, it may result in non-unique and/or unstable solutions^21^. The luck of uniqueness makes inverse problem difficult if not impossible in some cases^22^. For this reason, the inverse problem as usually encountered in unsaturated flow is said to be ill posed. In addition, the accuracy of predicted hydraulic properties depends on many factors such as the sampling design, the optimisation algorithm and the error in input data^20^. Knopman and Voss^23^ indicated that the accuracy with which unsaturated soil hydraulic parameters can be evaluated depends on points in space and time at which input data have been collected.

Most studies based on the development of inverse problem in subsurface hydrology have been restricted, for convenience, to homogeneous soil profile^16,20,24^. However, in several irrigated systems, the soil profiles are stratified as they may contain impermeable materials such as clay layer or gypsum obstacle^25^. In this case, there are two options to integrate the spatial variability of the soil characteristics in the model. The first option is to divide the soil profile into a number of homogeneous layers and the soil hydraulic properties are determined for each layer apart. The second is to apply a scaling method to characterise spatial variability in the soil hydraulic properties^26^.

The hysteresis phenomena between wetting and drying paths in soil water retention and hydraulic permeability curves is often overlooked, although it plays an important role in the water dynamics through the soil profile^27^, especially in the modelling of salinization processes induced by irrigation evaporation cycles. Nevertheless, considering this phenomenon induces additional parameters to identify and thus additional difficulties in the modelling and additional tests to undertake.

The main objective of this study was to identify the unsaturated hydraulic properties of a bare soil profile located in a Saharan Tunisian oasis. For this purpose, an inverse problem model was developed for determining the soil hydraulic properties from one-dimensional, transient ponded infiltration and internal drainage tests. Issues that are addressed include optimisation algorithms and effects of vertical stratification in the soil profile on the accuracy of identified hydraulic properties.

## Results

A ponded infiltration and an internal drainage tests were carried out in the bare soil profile selected for this study in the Segdoud oasis. Water contents and pressure heads were measured at different depths and at regular intervals. The infiltrated water amounts were also measured every 10 minutes during the infiltration test^28^.

### Inverse problem for homogenous soil

The inverse model developed in this work was applied to determine the unknown soil hydraulic parameters (θ_s_ porosity, K_s_ hydraulic permeability at saturation, α and n Van Genuchten functions parameters) for the experimental bare soil profile. On the first attempt, the soil profile was considered as a single homogeneous layer in order to simplify the problem. In this case, the infiltration and the internal drainage tests were simulated separately to determine the values of the unknown parameters and to highlight the hysteresis phenomena in the soil water retention and relative permeability curves. For each test, several simulations were performed.

Table 1 presents the specifications, set a priori, of four ponded infiltration test simulations. We vary the initial values of the optimised parameters (given in the second column) and RHOEND the final trust region radius^29^. The fourth column in Table 1 presents the formula used for the calculation of *J* the objective function, where *J*_θ_, *J*_ψ_, *J*_ϕ_ are root least square errors calculated with regard to, respectively, water contents, pressure heads and, infiltrated water fluxes.

**Table 1:** Specifications of the simulations during the ponded infiltration test.

(Assumption of homogeneous soil profile)			
Simulation	Initial Values n, α, K_s_, θ_s_	RHOEND	Calculation of J
1	3, 0.30, 1.04e-5, 0.3	1.e-2	J_θ_ + J_ϕ_
2	3, 0.25, 5.05e-5, 0.3	1.e-2	J_θ_ + J_ϕ_
3	3, 0.25, 5.05e-5, 0.3	1.e-2	J_θ_ + J_ψ_ + J_ϕ_
4	3, 0.30, 1.04e-5, 0.3	1.e-6	J_θ_ + J_ϕ_

Table 2 shows, for the four simulations listed above, the values of the objective function compounds (*J*_θ_, *J*_ψ_, *J*_ϕ_) at convergence. The low values of *J*_ϕ_ show that the surface inflows were well calculated. As the values of *J*_θ_ are smaller than those of *J*_ψ_, the measured volumetric water contents were better approached than the measured soil pressure heads. The variation of the initial values does not have much impact on the results. In simulation 4, the trust region radius (RHOEND) was reduced in order to obtain accurate results. However, no major changes in the results occur despite the increase in the number of iterations.

**Table 2:** Iteration number and objective functions values at convergence during the ponded infiltration test (Assumption of homogeneous soil profile).

Simulation	Iterations number	J_θ_	J_ψ_	J_ϕ_	J
1	56	0.762	2.657	0.0210	0.78
2	38	0.763	2.330	0.0209	0.78
3	36	0.948	1.868	0.0124	2.83
4	89	0.943	1.889	0.0096	0.95

The identified soil hydraulic properties for the four simulations are close enough (Table 3). The average soil porosity is equal to 0.21 cm^3^/cm^3^, n ≃ 2.9, α vary from 0.25 to 0.3 cm^−1^ and the average value of *K*_s_ is 2.6e^−5^ m/s. The simulation 2 provided the closest values of the soil hydraulic properties to the average ones.

**Table 3:** Identified parameters during the ponded infiltration test (Assumption of homogeneous soil profile).

Simulation	n	α (cm^−1^)	K_s_ (m/s)	θ_s_ (cm^3^/cm^3^)
1	3.99	0.340	2.80e-05	0.213
2	3.01	0.266	2.77e-05	0.212
3	2.93	0.270	2.50e-05	0.224
4	2.89	0.294	2.35e-05	0.212

**Figure 1:**
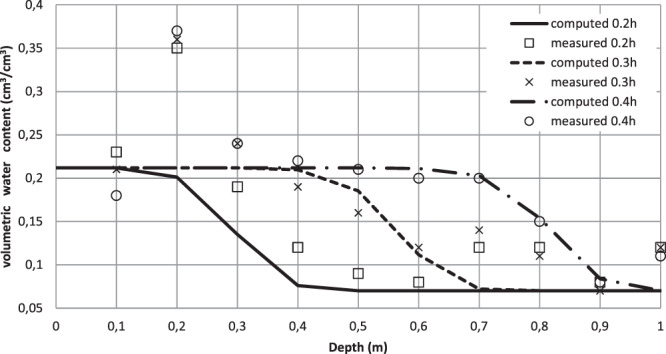
Measured and simulated (Simulation 2: *J = 0.78*) volumetric water content profiles during the infiltration test (Assumption of homogeneous soil profile).

The computed (simulation 2) and the measured volumetric water content profiles (at times 0.2 h, 0.3 h and 0.4 h from the beginning of the ponding) show good agreement except at depths of 20 cm and 75 cm (Fig. 1). In fact, at depth of 20 cm the measured water content reached 0.36 cm^3^/cm^3^ while it was less than 0,25 cm^3^/cm^3^ in the other depths. Larger water contents at this depth may be due to greater soil porosity. Silt and clay fractions in this depth may be higher than their contents in other soil layers. Therefore, the assumption of homogeneous soil profile is no longer acceptable.

The inverse problem model was used secondly to identify the soil hydraulic properties through the simulation of the internal drainage test, assuming a homogeneous soil profile. The computed soil pressure heads obtained at the end of the ponding were set as initial conditions. The objective function was calculated using the measured water contents, *J*_θ_, and pressure heads, *J*_ψ_. Several simulations have been carried out, the results of three of them are presented in Table 4. The average values of the soil hydraulic parameters are K_s_ = 4.4 e − 5 m/s; θ_s_ = 0.22 cm^3^/cm^3^, n = 2.4 and α = 0.15 cm^−1^. They are rather different from those given by the ponded infiltration test except the porosity value, which is the same. This difference is mainly due to the hysteresis phenomenon between drying and wetting paths, which results from pore shape irregularity.

**Table 4:** Simulation results of the internal drainage experiment (homogeneous soil profile).

Simulation	J_θ_	J_ψ_	n	α(cm^−1^)	K_s_(m/s)	θ_s_ (cm^3^/cm^3^)
5	1.55	6.49	3.282	0.327	8.09e-6	0.216
6	1.79	4.2	1.592	0.05	9.99e-5	0.224
7	1.59	6.9	2.593	0.15	2.52e-5	0.235

**Figure 2:**
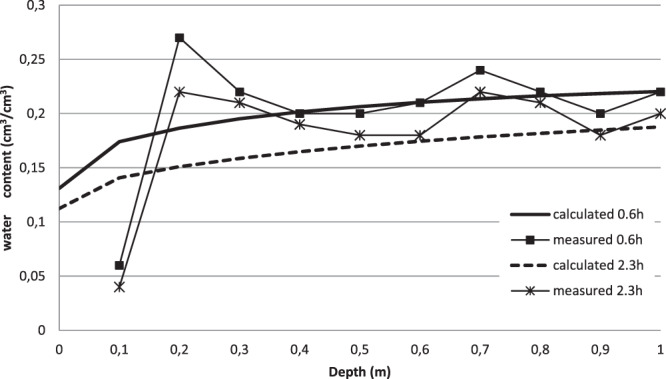
Measured and simulated (Simulation 7: *J = 1.59*) volumetric water content profiles during internal drainage test (Assumption of homogeneous soil profile).

Figure 2 illustrates the calculated (simulation 7) and the measured soil water content profiles during the redistribution stage. The comparison between these profiles at t = 2.3 h and at t = 0.6 h shows a general decrease of this variable due to the downward drainage. The simulated and the measured water content profiles have same shapes and trends. However, the water contents observed at 20 cm and 70 cm depths below land surface are higher than the computed ones. This discrepancy was previously recorded during the infiltration test. Globally, the model over-estimated the decreasing trend of the volumetric water content since the soil heterogeneity was not considered in the simulations (Fig. 2).

### Soil water retention and relative permeability curves

**Figure 3:**
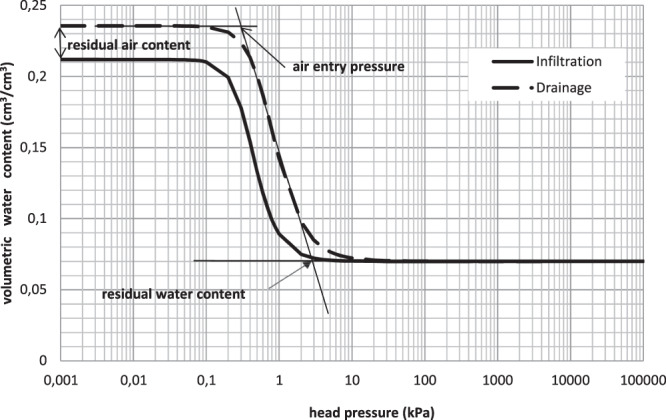
Calculated soil water retention curves for infiltration (simulation 2) and internal drainage (simulation 7) tests.

The hysteresis phenomenon was highlighted in the bare soil profile through the plotting of the soil water retention curves obtained during the ponded infiltration and internal drainage tests (Fig. 3). The curve corresponding to the drying phase is above the infiltration one due to effects of contact angle, trapped air, swelling and shrinking, and inkbottle effects^30^.

**Figure 4:**
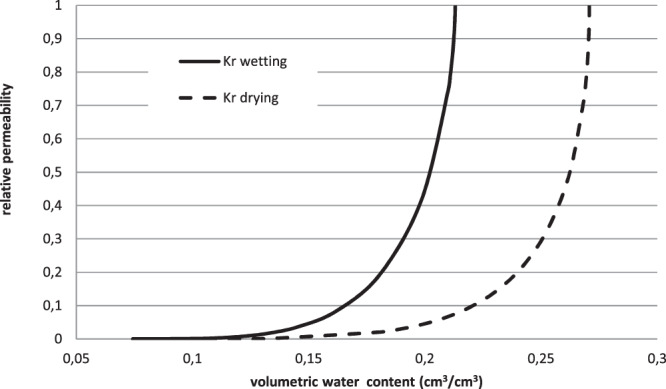
Calculated relative permeability curves during the wetting (simulation 2) and the drying (simulation 7) phases.

The relative permeability (kr) curves presents also two branches (Fig. 4). One branch corresponds to the wetting phase, the other one to the drying phase. For a given value of volumetric water content, the relative permeability in wetting phase is greater than that in drying phase. This is in agreement with the results presented by Van Genuchten^31^.

### Inverse problem for non-homogenous soil

The results presented in the above paragraphs showed that the experimental soil profile is not homogeneous as it was assumed. To overcome this limitation, the computing domain was subdivided into five layers and the inverse procedure was run in order to compute, simultaneously, the hydraulic parameters values for each layer. Several simulations of the infiltration test were conducted. Table 5 shows the results of two of them. In this table, θ_1_ is the saturated water content in the entire soil profile with two exceptions: at the layer around 0.2 m depth below land surface where the volumetric water content is θ_2_, and at depths 0.7 m and 0.8 m depths where the saturated water content is represented by θ_3_. Except the saturated water contents, the other parameters do not vary significantly from one layer to another.

**Table 5:** Simulation results of the infiltration test (Layered soil profile).

Simulation	J_θ_	J_ψ_	J_ϕ_	J	θ_1_	θ_2_	θ_3_
8	0.44	2.29	0.060	0.50	0.20	0.38	0.24
9	1.05	0.99	0.03	2.07	0.20	0.41	0.28

**Figure 5:**
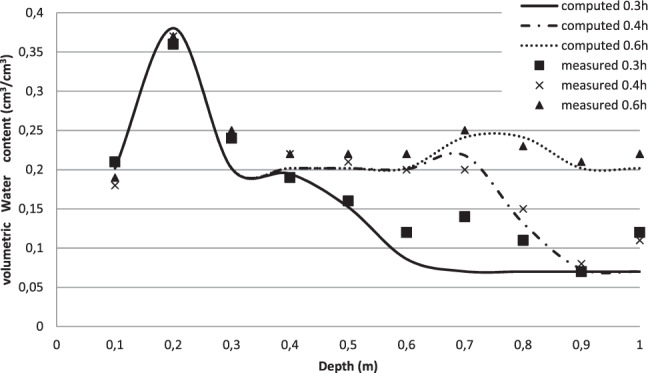
Measured and simulated (Simulation 8: *J = 0.5*) volumetric water content profiles during infiltration test (Layered soil profile).

Figure 5 shows a good concordance between the computed (simulation 8) and the measured volumetric water content profiles at the times t = 0.3, 0.4 and 0.6 h from the beginning of the ponded infiltration test. Our model simulated with a good accuracy the increase in the volumetric water content observed in the depth 0.2 m below the land surface. Furthermore, the decreasing trend of the water content when the soil depth increase from 0.2 to 0.3 m was also calculated with a good precision.

## Discussion

The values of the objective function compounds (*J*_*θ*_*, J*_*ψ*_*, J*_*ϕ*_) and the number of iterations at convergence were varying slightly with the variation of the initial values given to the unknown soil parameters (Tables 1 and 2). We already mentioned that this inverse problem is an ill posed problem^8^ and that the objective function is not monotonous. All these reasons suggest a non- unique solution to the problem. Nevertheless, BOBYQA algorithm used in this study was able to overcome the convergence problems and allows the identification of different sets of soil hydraulics parameters verifying the convergence conditions with about 38 iterations (no more than 89 iterations when increasing accuracy), so it demonstrated that it’s a rapid and reliable algorithm.

The objective function involving pressure heads, *J*_*ψ*_, has high values compared to *J*_*θ*_ and *J*_*ϕ*_. And unlike water content profiles, those of pressure heads are very different from measured pressure profiles. We noticed that the measured pressure heads remain negative all over the infiltration test. This can be explained by the inaccuracies of the tensiometers used for the measurement of pressure in unsaturated soil. In fact, these instruments can be disturbed by several environmental factors such as high air temperature and soil salinity^32^. Since Segdoud oasis is affected by soil salinization^2^, the measurements of the pressure heads may not be accurate. Therefore, the identification was focused on the minimization of (*J*_*θ*_, + *J*_*ϕ*_).

Several sets of parameters comply the convergence conditions. So which values should be adopted for this soil profile?

**Figure 6:**
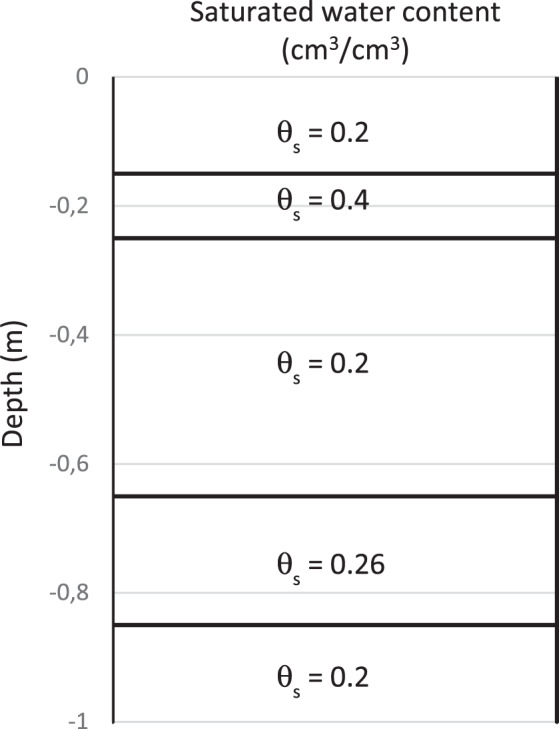
Saturated water contents of the layered soil.

It is well proven from the results that the soil profile contains one or more inclusions or stratifications. This spatial variability can be explained by the difference in soil texture. In fact, the sieve analysis performed in about 18 soils profiles located in the Segdoud oasis confirmed these results and showed that the soil in this ecosystem presents stratifications^28^. The spatial subdivision of the different layers as well as their saturated water contents, seem satisfactory regarding the results of Fig. 5. The adopted saturated water contents of the different soil layers are presented in Fig. 6.

The values of saturated hydraulic conductivity obtained by identification are close, the average is K_s_ = 2.6 e^−5^ m/s. It may be an appropriate value for the experimental bare soil profile, since it is the most determinant parameter in the calculation of the infiltrated water fluxes and *J*_*ϕ*_ is low in all simulations. This value is also within an acceptable range for a sand.

As the problem admits several solutions, we choose the most conventional and realistic values for the Van Genuchten parameters n and α. Considering the computing results and the sieve analysis, we adopt the following values:

-For the wetting path: n = 2.9 and α = 0.266 m^−1^

-For the drying path: n = 2.59 and α = 0.150 m^−1^

The residual air content in soil is obtained from soil water retention curve (Fig. 3) by subtracting the infiltration from the draining one as following:

1 $${\theta }_{s-draining}-{\theta }_{s-infiltration}={\theta }_{ar}$$

2 $$0.2355-0.2119=0.0236c{m}^{3}/c{m}^{3}$$

The air entry pressure is also deduced from the soil water retention curve (Fig. 3) and it is almost equal to 0.3kPa. The residual water content θ_r_ = 0.07 cm^3^/cm^3,^ as we initially set it.

In future work, these results can be compared with those obtained using other techniques such as the evaporation methods or the simplified analysis of internal drainage developed by Vauclin & Vachaud^10^.

The calculations have shown that the soil is layered. The model was able to reproduce correctly the flow through the different layers and to identify their parameters. However, preferential flows can occur at the interfaces between layers of different characteristics (Fine material / coarse material)^33,34^, or within wormholes or micro-fracture. If we take into account these phenomena (a dual-permeability model or modified Richards equation), the model would be more efficient but more complex and involving a large number of parameters^35,36^. Elaborating such model would be a good prospective for this work.

Since we are concerned with the salinization of Segdoud oasis, the identification of solute transport parameters is an obvious continuation of this work. It would require spatiotemporal measurements of soil salinity^18,24,26^.

## Materials and Methods

### Site description

**Figure 7:**
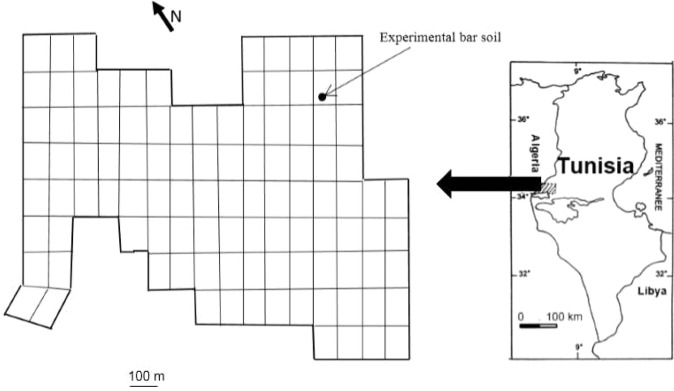
Location of the experimental bare soil in Segdoud oasis.

Segdoud oasis is situated in southwestern Tunisia (geographic coordinates are latitude 34°14′N and longitude 8°10′E) at about 60 km south of Gafsa city, in the catchment of *Chott El Gharsa* north (Fig. 7). The weather is hot, dry in summer and cold in winter. The average annual rainfall is less than 100 mm and the average annual evapotranspiration is more than 1400 mm^28^. Geological formations are mainly Quaternary alluvium deposits.

The oasis covers about 166 ha and is divided into 1.5 ha individual parcel of farmland. Each parcel is subdivided into regular basins (3 m × 3 m to 5 m × 5 m), limited by earth ridges that surround one date palm tree. Lands around basins are uncropped. The soil is classified as loamy sand to sandy (clay = 0%, loam ≤10%, sand ≥ 90%)^28^. Organic matter content is of about 2% in the superficial soil horizon^37^. The oasis contains three soil groups: arenosols in the median sector, calcic/gypsisols in the mounds of the meridional sector and slorchacks in lowlands^38^. Their spatial distribution depends on the geomorphology as follows:

Arenosols are poorly evolved coarse-texture alluvial soils. They can be salt-free or affected by salinization. The soil profile is homogenous sand with particular structure and very low organic matter content. It contains about 3 to 4% of gypsum.

Calcic/gypsisols may originate from the seasonal fluctuations of the shallow and saline water table^25^. They present a hard gypseous crust that is either apparent or submerged under a 20 cm depth layer of sand. Its gypsum content varies from 22 to 72%. The gypseous crust depth varies from few centimetres to 2.5 m below land surface.

Slorchacks have a sandy texture in surface (0–15 cm), sandy loam texture in subsurface (15–60 cm) and sandy texture in deeper horizon (60–140 cm). The medium soil salinity of these horizons are 17, 26 and 34 dS/m, respectively^38^. The salinization of these soils was induced by capillary rise process from saline and shallow water table^2^. These soils make up less than 5% of total oasis area.

### Field experiment

The experimental bare soil profile is located in the median sector of the oasis (Fig. 7). The soil texture is loamy sand relatively homogeneous with depth. The soil profile is free of hard gypseous crust; it allows the access of the neutron tube to the maximum depth of 1.20 m below the land surface. The water table was located at about 2.4 m depth; it enables the field calibration of the neutron moisture probe^39^. The experimental bare soil profile is representative of arenosols group. For the other two groups, the presence of hard gypseous crust and shallow water table don’t allowed the duplication of the tests.

A ponded infiltration and an internal drainage tests were carried out to determine the hydraulic properties of the experimental bare soil. The infiltration test was conducted using a double ring infiltrometer. The inner and outer rings diameters are 56 and 112 cm respectively. Four tensiometers were installed at 10, 30, 50 and 90 cm depths below land surface to measure pressure heads, and a neutron access tube was also installed to measure soil water contents at different depths. The two rings were flooded by a 3.0 cm water film, which was kept constant using a Mariotte’s bottle. The infiltrated water amounts were measured every 10 minutes by direct reading on the supply water tank. The water supply was stopped after 2 hours from the beginning of ponding. The total amount of the infiltrated water into the soil was about 127 cm. The soil surface was then covered by a plastic sheet once all the upper water film has disappeared to prevent evaporation and to allow the beginning of the redistribution of the infiltrated water (internal drainage test). The duration of this test was 21 hours. The volumetric water content was measured over the depth of 1.0 m below land surface with an increment of 10 cm. The soil water content and pressure head readings were made almost every 6 minutes during the infiltration test, and every 12 minutes to 4 hours during the internal drainage test.

### Model formulation

The unsaturated flow through the bare soil profile was described by the Richards equation written as follows:^40^

3 $$\frac{\partial \theta (h)}{\partial t}+div[-K(h)\overrightarrow{\nabla }(h-z)]=0$$

where:

*h* is the soil pressure head (L)

*θ* is the volumetric water content (L^3^/L^3^)

*K* is the soil hydraulic conductivity (L/T)

*t* is the time(T)

*z* is the vertical coordinate (positive upward)

The resolution of the Eq. (3) requires the determination of the initial and the boundary conditions as well as the soil hydraulic properties. The following initial condition was set:

4 $$h(z,0)={h}_{0}(z)$$

where *h*_0_ is the measured initial soil pressure head [L].

During the ponded infiltration test, the upper boundary condition was assumed a constant positive pressure head, which represents the thickness of the water film on land surface. During the internal drainage test, a nil flux was assumed at the land surface as the water film has disappeared and the evaporation process was avoided. The upper boundary conditions were set as follows:

5 $$h(0,t)={h}_{top}$$

for the ponded infiltration test

6 $${\rm{K}}({\rm{h}})\left(\frac{{\rm{dh}}}{{\rm{dz}}}-1\right)=0,\,z=0$$

for the internal drainage test

where *h*_top_ is the pressure head on land surface during the ponded infiltration test.

A free drainage was assumed at the bottom boundary since the water table was relatively deep. This condition was represented by a unit-gradient as follows^41,42^.

7 $${q}_{bottom}=-K(h)\frac{d(h-z)}{dz}=-K(h)$$

since

$$\frac{d(h-z)}{dz}=1$$

The downward water flux in the lower boundary, *q*_bottom_, is equal to the soil hydraulic conductivity, which is varying from zero when the soil is dry to *K*_s_ when the soil is saturated. In this case, the soil solution is allowed to pass freely.

### Soil hydraulic properties

The relationships between the volumetric water content and pressure head and between the soil hydraulic conductivity and pressure head were described using the following Mualem-van Genuchten functional relationships:^31,43^

8 $${S}_{e}=\frac{\theta (h)-{\theta }_{r}}{{\theta }_{s}-{\theta }_{r}}=\{\begin{array}{ll}{[1+{|\alpha h|}^{n}]}^{-m} & if\,h < 0\\ 1 & if\,h\ge 0\end{array}$$

9 $$K(h)={K}_{s}{S}_{e}^{l}{[1-{(1-{S}_{e}^{1/m})}^{m}]}^{2}$$

where:

*θ*_r_ is the volumetric residual water content(L^3^/L^3^)

*θ*_s_ is the volumetric saturated water content(L^3^/L^3^)

*K*_s_ is the saturated hydraulic conductivity (L/T)

α is the air entry parameter (1/L)

n is the pore size distribution parameter (-)

*l* is the pore connectivity parameter (-) which is always taken as 0.5^43^..

m is a soil parameter given by: m=1 - 1/n^43^.

### Water flow model

A cell-centred finite difference based model was developed using Matlab tool to solve the Eq. (3) in a vertical soil profile, with the pre-cited initial conditions (4), the boundary conditions (5), (6) and (7), and the functional relationships (8) and (9). The Eq. (3) was discretised with cells centred at the equidistant measurement locations. However, the numerical artefacts created by the use of a non-transparent numerical boundary condition for a free drainage do not allow a proper representation of the saturation front passage through the bottom boundary. Therefore, the simulated soil profile thickness was extended to 1.5 m.

### Inverse method formulation

The soil hydraulic properties (*θ*_r_, θ_s_, *K*_s_, *α* and *n*) for the studied bare soil profile are unknown. The value of *θ*_r_ was fixed a priori to 0.07 cm^3^/cm^3^ since it is the least sensitive parameter to the most of models calibration^44^. An inverse problem method was developed for the estimation of the other hydraulic properties. This method compares the measured infiltrated water flux, volumetric water contents and pressure heads to those calculated by the numerical model solving the Richards equation. The objective function, *J*, used for this comparison is the sum of three terms corresponding to the three measured variables:

10 $$J(A,X)=\mathop{\sum }\limits_{i=1}^{3}{J}_{i}(A,X)$$

where: ${J}_{i}(A,X)$ is the root least square error, calculated as follows:

11 $${{J}_{i}(A,X)={W}_{i}{A}_{i}^{c}(X)-{A}_{i}^{\ast }\Vert }_{2}^{2}$$

and:

${A}_{i},i=1,2,3$ are respectively the water contents, the pressure heads and the infiltrated water fluxes.

${A}_{i}^{c}(X)$ are the computed values,

${A}_{i}^{\ast }$ are the measured values,

$X=({K}_{s},n,\alpha ,\Phi )$ is the vector of the optimized parameters,

${W}_{i}={\left(\frac{1}{max({A}_{i}^{\ast })-min({A}_{i}^{\ast })}\right)}^{2}$ are the weights associated to measurements.

The three components of the objective function are the followings:

${J}_{1}(A,X)={J}_{\theta }$ with regard to water contents,

${J}_{2}(A,X)={J}_{\psi }$ with regard to pressure heads,

${J}_{3}(A,X)={J}_{\phi }$ with regard to infiltrated water fluxes.

The optimisation problem consists in finding the vector X(θ_s_,K_s_,n,α) that minimises the objective function J(A,X).

### Optimisation algorithm

**Figure 8:**
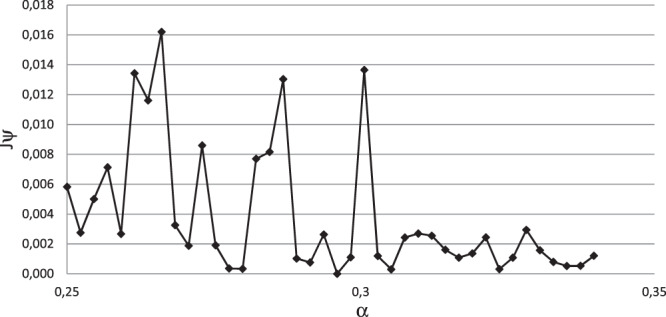
Objective function *J*_*ψ*_ sensitivity to parameter α variation.

Since the inverse problem for unsaturated water flow is an *ill posed* problem^8^, the values of the soil hydraulic properties are non-unique. Three optimisation methods were tested in order to resolve this problem. The first method is the Matlab gradient-based optimising function, in which both the objective and the constraint functions should be continuous and yields local solution. As *J*_*ψ*_ of this problem is a non-monotonic function of n and α (it is actually very unstable and has several local minima as shown in Fig. 8), the optimum set of unknown properties could not be found using this method. The second one is the Particle Swarm Optimization algorithm, which is based on Swarm Intelligence techniques. This algorithm was initially proposed to simulate social behaviours^45^. It produces quite good results but takes long computation time as it calculates 2020 time the objective function (and as many times the direct problem). The third one is the Bound Optimisation BY Quadratic Approximation (BOBYQA).This is an iterative algorithm seeking the least value of an objective function whose parameters are subject to simple bounds^29^. The particularity of this algorithm is that no first derivatives of the objective function are required explicitly. It was retained in this study to minimise the objective function.

### Model validation

**Figure 9:**
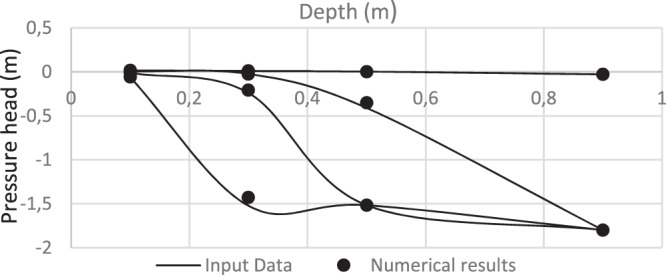
Given and identified pressure heads profiles (Validation test).

The direct problem model was tested using the Philip solution^46^. It gives suitable results. The inverse procedure was also validated using data of simulated (synthetic) infiltration experiment. The identified parameters are close to the input data (Table 6). Figure 9 shows that the computed pressure heads values match the given pressure heads profiles, at different times from the beginning of the infiltration test. This demonstrates that our model is able to give good results.

**Table 6:** Input data and identified parameter for a simulated infiltration experiment.

	K_s_	θ_s_	α	n
Input Data	4.e-5	0.33	0.296	3.5
Numerical results	3.98 e-5	0.33	0.296	3.67
Relative error	0.005	0	0	0.05

## Conclusion

The inverse problem method developed in this study has allowed the determination of the soil hydraulic properties in a bare soil located in the Segdoud oasis. The data used for this work were collected *in situ* and they reflect the real condition of flow processes occurring in this ecosystem. In this work, we tried to identify the best optimisation algorithm to avoid the problem of non-unique and/or unstable solutions generally encountered when using this method. The retained algorithm BOBYQA is efficient and rapid. Further, we developed a simple technic to overcome the problem of soil hydraulic properties identification when the soil profile contains one or more stratifications. Furthermore, we highlighted the phenomena of hysteresis in the experimental soil profile through the plotting of the water retention curves for wetting and drying paths. The set of soil hydraulic parameters determined in this study can be used to simulate efficiently the irrigation scenarios that would reduce the soil salinization in this vulnerable ecosystem. Moreover, the model developed in this work is simple and efficient, it could be used for other soils and in other studies.
